# Neurostimulation with Naming Therapy for Primary Progressive Aphasia: A Pilot Study Targeting Transcranial Direct Current (tDCS) Stimulation for the Individual

**DOI:** 10.3390/brainsci16020128

**Published:** 2026-01-25

**Authors:** Christopher Bernard Leahy, Jennifer C. Thompson, Matthew Jones, Anna Woollams

**Affiliations:** 1 The Northen Care Alliance NHS Foundation Trust, Salford M6 8HD, UK; jennifer.thompson@manchester.ac.uk (J.C.T.); matthew.jones@nca.nhs.uk (M.J.); 2 Division of Psychology, Communication and Human Neurosciences, School of Health Sciences, Faculty of Biology, Medicine and Health, University of Manchester, Manchester M13 9PL, UK; anna.woollams@manchester.ac.uk; 3 Division of Medical Education, School of Health Sciences, Faculty of Biology, Medicine and Health, University of Manchester, Manchester M13 9PL, UK

**Keywords:** PPA, tDCS, speech and language therapy, neurostimulation, home-based tDCS

## Abstract

**Background**: Transcranial Direct Current Stimulation (tDCS) in conjunction with behavioural language therapy in PPA has previously been modified for variation at the group level, but not at the individual level. This pilot study used individualised tDCS targeting by identifying regions of peak atrophy in the language system. **Methods**: Six PPA participants (four semantic and two non-fluent variant) were randomly allocated to receive tDCS or sham stimulation. The target electrode was selected for each based on their region of peak atrophy. Participants received naming therapy, individually calibrated according to baseline naming performance. Three sets of therapy were delivered in conjunction with tDCS (1 mA) or sham stimulation within participants’ homes. The study was not powered to demonstrate efficacy but to show proof-of-concept for an individualised, home-based tDCS targeting method. **Results:** All participants successfully completed the protocol. In one participant the region of peak atrophy differed from that predicted by clinical syndrome. Significant gains were observed at an individual level for treated items in both groups (2/3 tDCS and 2/3 Sham). No significant changes in untreated items were observed at an individual level. Significant naming improvement in untreated items was not observed for the tDCS group and was seen at one time point only for the Sham group. **Conclusions**: We have demonstrated the feasibility of a novel method for selecting neurostimulation targets for PPA at the individual level. A larger study would be required to determine the long-term therapeutic efficacy of this method.

## 1. Introduction

Transcranial Direct Current Stimulation (tDCS) is a technique that produces a weak electrical current over the scalp to stimulate the cortex. tDCS has been evaluated extensively as a treatment for stroke aphasia. Left hemisphere stimulation has shown promise as a therapeutic intervention for stroke in isolation [1,2] but more commonly as an adjunct to language therapy [3,4,5,6,7,8]. A perilesional approach, whereby the primary site of damage within the language network is stimulated [3,5] has typically been employed.

Primary progressive aphasias (PPAs) are neurodegenerative syndromes characterised by progressive loss of language function in the absence of the deficits in memory, visuospatial, or behavioural function seen in other forms of dementia [9,10]. PPA is divided diagnostically into three variants: nonfluent (nfvPPA), logopenic (lvPPA), and semantic (svPPA) [11]. There also exists clinical variation within these canonical groups and some PPA cases do not conform to a single subtype. There are no currently available disease-modifying treatments for PPA, making the development of symptomatic forms of management important, which has mainly included the use of speech and language therapy.

tDCS has received increasing research attention as a tool for the symptomatic management of PPA [12]. There is growing evidence that combining tDCS with language therapy may be symptomatically beneficial, including enhancement of generalisation of gains to untreated items within nfvPPA [13,14], svPPA [15], and mixed populations that include lvPPA patients [16,17]. Despite this promise, there are a number of methodological issues in relation to the heterogeneity of PPA that need to be resolved to plan large scale trials.

Selection of a sub-optimal stimulation target has been highlighted as a factor that could limit therapeutic gains in stroke aphasia [18]. PPA syndromes are associated with distinct atrophy patterns at the group level. However, despite these recognised patterns of atrophy, demonstrated by imaging studies [19,20,21,22], variation in atrophy patterns between individuals with similar clinical features is observed; for example, one study found that 44% of nfvPPA participants with clinically supported criteria did not demonstrate radiologically typical features [23]. Problems in predicting the site of atrophy from diagnostic label may result in failure to select the optimum target for tDCS, thus reducing therapeutic efficacy. The known heterogeneity of atrophy patterns in PPA means that therapy may need to be individualised.

This pilot study aims to address the feasibility of a personalised approach to tDCS targeting, selecting language areas closest to peak atrophy for stimulation as a home-delivered approach in conjunction with speech and language therapy to improve naming. The goal of this study is to demonstrate proof-of-concept of an imaging-driven, individualised method for neurostimulation targeting.

We identified the following aims:

• Provide home-based, individualised tDCS-augmented speech therapy to people with PPA;
• Individualise both the speech therapy component and the stimulation electrode placement (using participant’s individual MRI brain atrophy patterns);
• Pilot a study design that could be scaled up for a larger trial of individualised tDCS in PPA.

## 2. Materials and Methods

### 2.1. Participant Identification and Recruitment

Participants were recruited from a tertiary cognitive neurology clinic and met the following criteria: fulfilling standardised diagnostic criteria [11] for non-fluent (nfvPPA), semantic (svPPA), or logopenic variant (lvPPA) PPA, right-handed, and with clinically significant naming impairment but not dense anomia, defined by Graded Naming Test (low frequency nouns) score <14 and Manchester Naming Test (locally developed naming test comprising 40 high frequency nouns, [24]) >15. Patients receiving a new diagnosis of PPA were screened for eligibility within a fixed time window (December 2022–June 2023). Participants and their care partners who gave consent to be contacted to discuss the study were provided with study leaflets and contacted by telephone to discuss the study. The consent process was then finalised at a visit to the patients’ homes at least one week after first contact. All patients receiving a new diagnosis of PPA within the recruitment window were screened. Eight participants met the inclusion criteria and were contacted, of whom, six elected to participate and were recruited. This small sample size was designed to demonstrate the feasibility of our method rather than therapeutic effectiveness. Their demographics, PPA-subtype, naming scores, and selected cognitive measures (obtained as part of their standard diagnostic work-up) are summarised in Table 1. The assessment tools included in Table 1 are part of a standardised assessment battery administered for diagnostic purposes in the cognitive neurology clinic from which participants were recruited. These have been provided to illustrate the overall neuropsychological profile of the recruited study participants.

### 2.2. Individualised Speech Therapy Approach

All testing and therapy took place in the patients’ homes. To assess baseline naming, participants completed a 407-item naming test on two separate occasions, consisting of items from the International Picture Naming Project (IPNP) [25]. Items named incorrectly twice were used to generate two sets: “incorrect treated” to evaluate the effect of therapy, and “incorrect untreated” to assess generalisation to untrained items. Items named correctly twice were included in an additional set of “correct untreated” items to monitor progression of the naming deficit over time. Each of these 3 sets consisted of 32 items, based on set sizes used in previous intervention studies of PPA shown to yield lasting benefits [26], which were matched across sets for name agreement, phonemic and syllabic length, and frequency (assessed by a between-group t-test with a *p* value of 0.05 on each variable).

An individualised naming therapy PowerPoint slide deck was then created for each participant comprising their 32-item “incorrect treated” set. A multimodal naming therapy previously employed for stroke aphasia with tDCS [27] was used, in which a colour photograph representing the relevant IPNP object was presented on a laptop screen together with an audio–video clip of a mouth naming the item. During the naming therapy protocol, participants viewed the slide and were prompted to repeat back the item name, followed by a 2 s automated delay before presentation of the next slide. Each 32-item set was repeated in randomised order 10 times during each therapy session.

### 2.3. Process for Identifying the Region of Peak Atrophy Within the Language Network

To identify the region for neurostimulation, we developed a method for identification of peak regional atrophy on T1 imaging using SPM 12, with reference to a healthy control imaging dataset obtained from previous studies by the research group. This dataset consisted of 19 right-handed healthy adults (8 females, 11 males) with a mean age of 68 years (standard deviation = 6, range = 59–80).

T1 images were segmented and normalised using a unified procedure implemented in SPM12 software package in MATLAB software [28]. Images were smoothed with an 8 mm kernel applied for normalised grey matter and white matter segments. From this, a voxel was assigned a value based upon the probability of grey matter (GMPV) for all images.

The mean GMPV was calculated for controls for each voxel. The standard deviation of the control GMPV was calculated for each voxel, multiplied by two, and subtracted from the mean control GMPV for each voxel to assign a threshold of abnormality. Subject voxels with a GMPV less than this threshold value of abnormality were calculated and binarized to illustrate atrophy within a Binary Abnormality Map (BAM). A mask (a highlighted region obtained from the MRICron Brodmann Atlas) was applied for all Brodmann Areas (BA) within all cortical regions with language function within the left hemisphere. The Brodman Atlas was chosen because these areas align well with PPA-specific anatomy. In addition, as a widely used atlas, it improves cross-study comparability. This mask included BA 4, 6, 8, 20, 21, 22, 37, 38, 39, 40, 44, 45, and 47 taken from the MRICron. We eliminated Brodmann Area 25 in the inferior–medial frontal lobe from our analysis as this region was not viable as a target for electrical stimulation via the scalp. The BAM was used to calculate abnormal voxels and represented as a percentage of total voxels per BA. The BA with the highest percentage of abnormal voxels was considered the BA with peak atrophy (Peak BA).

To achieve a perilesional approach to neurostimulation, we identified the immediately contiguous region to the Peak BA with the lowest abnormality value: this ‘Target Brodmann Area (BA)’ was used as the target stimulation site. The rationale for selection of this adjacent BA was to avoid stimulating the site of most severe atrophy, which may limit the tDCS response due to neurones failing to achieve stimulation threshold [18]. From this, a ‘Target Electrode’ within the ‘10–20’ electrode system was selected. This was the electrode either within, or in closest proximity to, the Target BA based upon MNI coordinates [29]. Where target electrodes were equidistant to a target BA, the electrode position closest anatomically to the Peak BA was selected for neurostimulation.

### 2.4. tDCS or Sham Stimulation with Naming Therapy Protocol

Participants underwent three sessions of tDCS or sham stimulation on separate days within one week, delivered in their homes. The anodal stimulation condition involved the application of 25 min of tDCS at 1 mA, while for the sham condition, anodal stimulation was administered for 30 s before being slowly turned off. This sham procedure has been verified experimentally previously in stroke aphasia and healthy patients [30]. The previously described individualised naming therapy was administered in 10 sets of 32 items during either tDCS or sham stimulation.

### 2.5. Outcomes of Interest

#### 2.5.1. Individualised tDCS Applications (i.e., Electrode Placement)

The outcome of our imaging-derived choice of target electrode was compared with a target based upon the patient’s clinical syndrome.

#### 2.5.2. Naming Therapy-Based Measures (Treated vs. Untreated Items)

Naming ability was assessed before, immediately after, one day after, and again a further six weeks after therapy for the 32 incorrect treated, incorrect untreated, and correct untreated items. The incorrectly named treated items were included to show therapeutic benefit on naming; incorrectly named untreated items were included to show generalisation of therapeutic effect; the set of items named correctly before therapy were included to demonstrate any deterioration from baseline. The primary outcome measure was change in percentage naming accuracy from pre-therapy to post-therapy.

#### 2.5.3. Quality of Life Measures

A secondary outcome was a measure of functional communication that has shown improvement in previous studies combining speech and language therapy with tDCS in stroke aphasia [31], the Stroke and Aphasia Quality of Life Scale (SAQOL-39) [32], which was completed by the person with PPA. This was administered before therapy, one day after, and six weeks after completion of the naming therapy. This scale has been included within consensus-based recommendations for core outcome sets in PPA research [33] and shares common constructs with other validated quality of life measures [34].

#### 2.5.4. Data Analysis

Change in naming score from baseline at an individual and group level was assessed using Wilcoxon signed rank tests. Nonparametric tests were adopted due to the small sample size, which makes it difficult to reliably assess the normality of the distribution [35].

## 3. Results

### 3.1. Feasibility of Study Protocol

All six patients who consented to the study protocol completed all aspects of therapy and follow up of primary outcomes. The method to identify peak atrophy and select a target electrode was successful for all participants.

### 3.2. Individualised tDCS Application

The four svPPA participants had a location of peak atrophy at the anterior temporal lobe and a target electrode of T3. One nfvPPA participant also showed a pattern of peak atrophy at the anterior temporal lobe with a target electrode of T3. The other nfvPPA participant showed peak atrophy in the superior frontal gyrus, a typical region of involvement for this subtype, with a target electrode of C3. Table 2 summarises tDCS targeting.

**Table 2:** Summary of tDCS targeting.

Participant	Variant	Peak BA	Target BA	Target Electrode	Typical Target Electrode
tDCS 1	nfvPPA	8	6	C3	C3
tDCS 2	svPPA	38	22	T3	T3
tDCS 3	svPPA	38	22	T3	T3
Sham 1	svPPA	38	22	T3	T3
Sham 2	svPPA	38	22	T3	T3
Sham 3	nfvPPA	38	22	T3	C3

Peak BA—region of peak atrophy from the MRI data; Target BA—adjacent target region identified from MRI data; Target Electrode—subsequent electrode position generated; Typical Target Electrode—the target electrode that would have been selected based upon typical atrophy for that syndrome.

### 3.3. Naming Therapy Outcomes

#### 3.3.1. Treated vs. Untreated Outcomes in Overall Cohort

**Figure 1:**
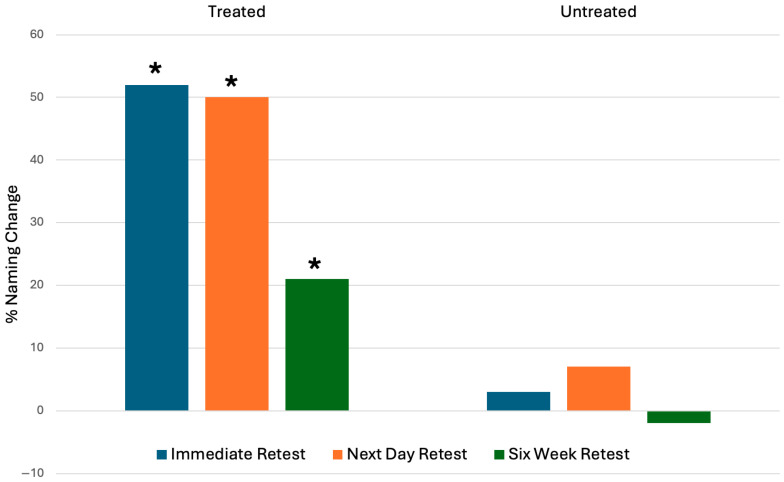
Overall group naming score change calculated as a percentage of incorrect items at baseline at immediate retest, next day retest, and six-week retest for treated vs. untreated items (* denotes significant change from baseline (*p* < 0.05) on Wilcoxon signed rank test).

At an overall group level, PPA patients demonstrated significant improvement in naming compared to baseline for treated items at all time points, as shown in Figure 1.

Naming scores were significantly higher for treated items compared to baseline at immediate retest (z = 9.39 *p* < 0.001), next day retest (z = 8.89 *p* < 0.001), and six-week retest (z = 4.87 *p* < 0.001). Untreated item naming scores were significantly higher compared to baseline at one time point: next day retest (z = 2.2 *p* < 0.28). Items correctly named at baseline did not demonstrate a significant deterioration over the time points.

#### 3.3.2. tDCS vs. Sham Stimulation Outcomes

**Figure 2:**
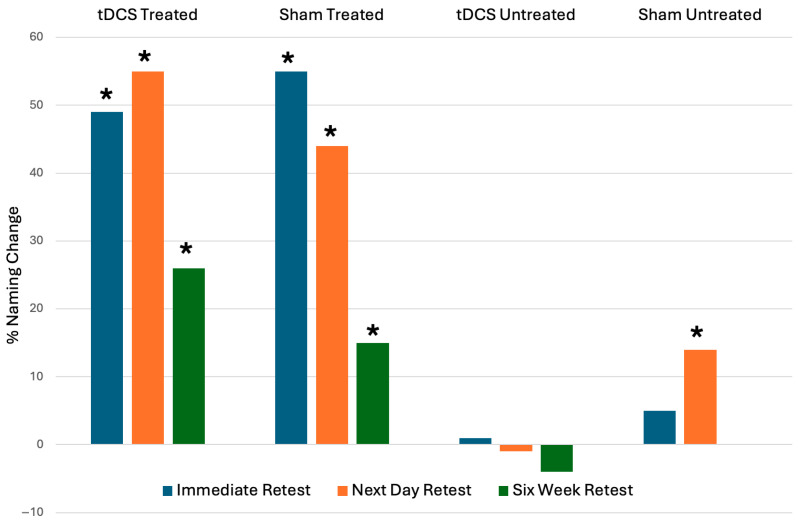
Naming score change calculated as a percentage of incorrect items at baseline at immediate retest, next day retest, and six-week retest for tDCS vs. Sham treated and untreated items (* denotes significant change from baseline (*p* < 0.05) on Wilcoxon signed rank test).

As a group, the tDCS naming scores were significantly higher for treated items compared to baseline at immediate retest (z = 6.58 *p* < 0.001), next day retest (z = 7.02 *p* < 0.001), and six-week retest (z = 4.35 *p* < 0.001). The sham group showed significantly higher naming scores for treated items compared to baseline at immediate retest (z = 6.72 *p* < 0.001), next day retest (z = 5.59 *p* < 0.001), and six-week retest (z = 2.6 *p* = 0.009). The sham group also demonstrated significant gains for untreated items at next day retest (3.34 *p* < 0.001). Changes in the number of items named, calculated as a percentage of incorrect items at baseline, are shown in Figure 2.

#### 3.3.3. Individual Response to Naming Therapy

**Figure 3:**
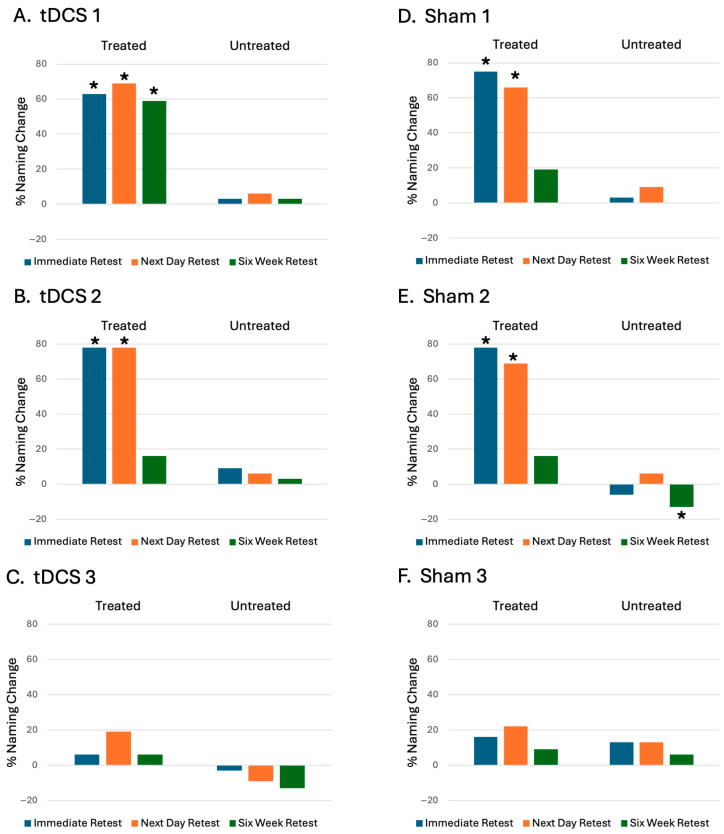
Naming score change, calculated as a percentage of incorrect items at baseline, at immediate retest, next day retest, and six-week retest, for individual participants: (**A**). tDCS 1, (**B**). tDCS 2, (**C**). tDCS 3, (**D**). Sham 1, (**E**). Sham 2, (**F**). Sham 3 (* denotes significant change from baseline (*p* < 0.05) on Wilcoxon signed rank test).

All participants showed an improvement in naming scores for treated items from baseline. There was no overall pattern between baseline performance and therapy outcome. Changes in the number of items calculated as a percentage of incorrect items at baseline for individual participants are shown in Figure 3. Statistically significant gains were observed at an individual level for treated items for two of the tDCS participants: a nfvPPA participant (tDCS 1) at all time points, and a svPPA participant (tDCS 2) at immediate retest and next day time points; for two sham svPPA participants (Sham 1 and Sham 2) at immediate retest and next day time points.

One sham svPPA participant (Sham 2) showed a significant reduction in untreated items at six weeks. Otherwise no statically significant changes were seen for untreated items.

The tDCS participant who showed the least gains for treated items (tDCS 3) demonstrated a deterioration in correct items, illustrating a deterioration from baseline at all time points.

### 3.4. Quality of Life

The participant self-completed quality of life questionnaire (SAQOL-39) demonstrated internal consistency (>0.9 Cronbach’s alpha) and showed no significant change from baseline for tDCS or sham groups, apart from one psychosocial score which demonstrated a significant improvement from baseline 6 weeks post therapy in the tDCS group. This was an overall psychosocial score included in the SAQOL-39 referring to activities of daily living and engagement in social life. This showed an improvement from 66% performance at baseline to 73% (*t* test *p* < 0.05).

## 4. Discussion

This study piloted an individualised method of tDCS-enhanced language therapy. This included personalised naming sets, created as part of each participant’s therapy, alongside the use of MR imaging to identify peak atrophy, and tDCS electrode targeting at an individual level. All participants completed the protocol, which was delivered in the home environment with no adverse events.

Using an individualised imaging-based approach, we identified one participant in which the region of peak atrophy differed from that predicted by their clinical syndrome. In this case, a nfvPPA participant unexpectedly demonstrated peak atrophy at the anterior temporal lobe. This case demonstrates the potential benefit of a personalised approach to tDCS targeting if aiming for perilesional stimulation.

Alongside variability in atrophy patterns for individuals with the same PPA subtype diagnosis, we observed variability in therapy response at the individual level. Such variation in treatment effect with neurostimulation has been noted within previous studies [18,36].

Whilst most participants saw improved naming measures after therapy, two individuals did not demonstrate this effect, even for treated items (tDCS-3 and Sham-3). tDCS-3, a svPPA participant, was the youngest participant and performed relatively poorly on baseline naming and fluency tests during their diagnostic assessment compared to the other two participants receiving tDCS. Previous neurostimulation studies in PPA that have analysed naming at an individual level have not found reliable patterns of therapy response based upon baseline naming performance [37]. In our small cohort, there was also no overall pattern between baseline performance and therapy outcome. tDCS-3 also demonstrated a higher proportion of atrophy in her peak language BA, the left anterior temporal lobe, than the other tDCS participants. Severity of atrophy has been previously proposed as a factor limiting tDCS response due to a higher proportion of neurones failing to achieve a therapeutic stimulation threshold [36]. Sham-3, the other participant showing no significant response to therapy, had nfvPPA and was the oldest recruited. The proportion of abnormal voxels within his most atrophied region, the left anterior temporal lobe, was also higher than all-but-one of the other cases recruited. This participant demonstrated the most severe overall cortical atrophy of the participants.

Another factor known to affect response to this type of therapeutic intervention is PPA variant. Challenges in achieving generalisation to untrained items have been identified in svPPA therapy studies both with [16,36] and without neurostimulation [38]. In our small cohort svPPA was overrepresented, although the individuals who seemed to respond worse included both a svPPA and a nfvPPA participant. It is, however, notable that this nfvPPA participant had peak atrophy in the anterior temporal lobe region. Therapeutic limitations might be predicted in svPPA given the critical importance of semantic processing for naming tasks. Other studies of svPPA that managed to show an effect for trained and untrained items at the group level incorporated either a semantic-focused therapy [15], or a more flexible therapeutic approach, whereby the focus was adapted to a more semantic or phonological approach based upon the encountered deficits [39]. We used a common computerised multimodal naming therapy for all participants. Our approach individualised naming therapy based upon incorrectly named items by a participant. This sought to address deficits common to, and identified early within, all three canonical PPA variants. However, this method was developed in chronic stroke aphasia for participants with non-fluent or anomic deficits with relative retention of semantic function and may have a more limited role outside of this setting. Future studies aiming to individualise tDCS to account for heterogeneity of brain atrophy might also benefit from the use of different language therapies that address the underlying causes of naming deficits.

### 4.1. Study Limitations

This small pilot study was not powered to detect differences between tDCS and sham groups, but nevertheless the absence of any clear signal for neurostimulation efficacy warrants discussion. We adopted a perilesional approach that targeted areas in proximity to peak regions of atrophy for neurostimulation. Our method depended upon relative, rather than absolute, evaluation of atrophy to select a contiguous target BA with the purpose of selecting a cortex sufficiently intact to respond to stimulation. It is possible that the relatively spared, contiguous language regions selected were still too significantly involved to be amenable to stimulation that augments naming therapy interventions. There have, however, been previously encouraging outcomes when stimulating perilesional regions of high disease involvement, such as left anterior temporal lobes for svPPA participants [15] and the inferior frontal lobe in nfvPPA [13,14,40]. Optimum placement of tDCS electrodes remains an area for further research in larger scale studies.

Notwithstanding the perilesional targeting debate, there are potential issues related to the use of Brodmann areas for the purpose of choosing neurostimulation sites. The size and borders of BAs do not directly map onto electrode sites within the 10–20 EEG system. Despite attempts to account for variable size of these areas by calculating the proportion of abnormal voxels, larger BAs may under-represent highly concentrated regions of atrophy due to inclusion of relatively uninvolved cortex. The morphology of BAs is not uniform; this leads to issues when selecting the electrode closest to the target BA. For example, narrow segments within a BA including fewer abnormal voxels may extend near a target electrode and lead to its selection, despite most of the BA’s abnormal voxels lying more remote to this position.

Further limitations of our methodology relate to our population recruitment strategy, which identified an imbalanced PPA population in terms of the subtypes included (svPPA predominant). This limits the generalisability to future studies for other forms of PPA. In addition, the tDCS manipulation was conducted between participants, in an effort to avoid potential carryover effects and progression during the course of treatment that would have arisen using a within-participants design. Given the individual variability observed within PPA, this between-participants design necessarily reduced power to detect any significant tDCS effects. Furthermore, maintenance of language gains was demonstrated at short-term follow up but not assessed over a longer duration, diminishing conclusions with respect to the longevity of the observed treatment benefits.

### 4.2. Future Directions

To enhance outcomes, future studies using tDCS for PPA may choose to consider within-group heterogeneity and how this may be accounted for. An inherent issue for study protocols is how best to select tDCS targets at the individual level. Our pilot demonstrates the viability of an imaging-driven approach to address this based upon atrophy. Future research may wish to adopt our individualised stimulation site approach based upon strengths of our method, including reproducibility using a standardised brain atlas, balance between pathological involvement and structural integrity, and application in patients’ homes [41]. Given the methodological limitations we have acknowledged and the diversity of imaging modalities available, future studies may wish to modify our protocol. However, given our findings, studies should justify how they may account for such differences to enhance and maintain their language outcomes.

In our study, we used a combination of semantic and articulatory cues to repetition as a treatment for anomia for all cases, irrespective of subtype, in order to reduce variability via intervention consistency. Future research could explore the use of language therapies more tailored to a participant’s deficits. For example, those with svPPA may benefit more from an intervention based on semantic feature analysis [42] or semantic matching [15], whereas those with nfvPPA or lvPPA may benefit more from a focus on phonological manipulation and transcoding [43]. For mixed PPA samples, error types can be used to determine whether intervention should involve semantic or phonemic cues [16,36]. In addition, future research could consider other language abilities apart from object picture naming, such as verb naming [17], spelling [44], and short-term/working memory [45]. Given the linkage observed between inner speech and aphasia recovery, it is also possible that tDCS of areas such as the left inferior frontal gyrus may bolster residual inner speech capacities to enhance therapeutic outcomes in PPA [46].

## 5. Conclusions

In summary, our imaging-driven method for selecting tDCS targets was piloted in conjunction with personalised naming therapy for six PPA participants in their own homes. Our approach was found to be feasible and resulted in a different choice of neurostimulation site for one participant in comparison with a conventional syndrome-driven selection method. Therapy resulted in naming improvement for both treatment arms and in one active tDCS participant, this was maintained at the six-week follow up. There was no demonstrable difference between tDCS and sham stimulation within this small sample of svPPA and nfvPPA participants. More evidence is required to fully assess the utility of our individualised targeting approach, but given the successful demonstration of feasibility, this could be scaled up in a larger study.

## Figures and Tables

**Table 1:** Participant characteristics.

Participant	PPA Variant	Age	Symptom Duration (yrs)	MMSE	F-A-S	DFS	MNT	GNT
tDCS 1	nfvPPA	64	3	25	21	5	37	9
tDCS 2	svPPA	54	4	30	34	7	38	3
tDCS 3	svPPA	51	1	26	17	6	29	1
Sham 1	svPPA	65	4	28	72	8	39	3
Sham 2	svPPA	73	3	28	39	5	21	0
Sham 3	nfvPPA	81	2	26	30	7	36	9
Mean (sd)	n.a.	65 (11)	3 (1)	27 (2)	36 (20)	6 (1)	33 (7)	4 (4)

MMSE—Mini Mental State Examination; F-A-S—fluency test; DFS—Digit Forward Span; GNT—Graded Naming Test; MNT—Manchester Naming Test. The F-A-S fluency test assesses phonemic verbal fluency by asking an individual to orally produce words beginning with letters F, A, and S. The Digit Span requires subjects to repeat a series of digits of increasing length, testing verbal short-term memory.

## Data Availability

The raw data supporting the conclusions of this article will be made available by the authors on request.

## Abbreviations

The following abbreviations are used in this manuscript:

PPA	Primary Progressive aphasia
tDCS	Transcranial Direct Current Stimulation
nfvPPA	Nonfluent PPA
lvPPA	Logopenic PPA
svPPA	Semantic PPA
BAM	Binary Abnormality Map
